# Supplementary material for: Cytotoxic Properties of HT-2 Toxin in Human Chondrocytes: Could T3 Inhibit Toxicity of HT-2?
Source: Toxins (Basel). 2019 Nov 15;11(11):667. doi: 10.3390/toxins11110667 (PMC6891367; doi:10.3390/toxins11110667)
Supplement: Supplementary file 1 [file toxins-11-00667-s001.pdf]

Toxins **2019**, *11*, x; doi:

Supplementary Materials: Cytotoxic Properties of HT-2 Toxin in Human Chondrocytes: Could T3 Inhibit Toxicity of HT-2?

Feng'e Zhang, Mikko Juhani Lammi, Wanzhen Shao, Pan Zhang, Yanan Zhang, Haiyan Wei and Xiong Guo

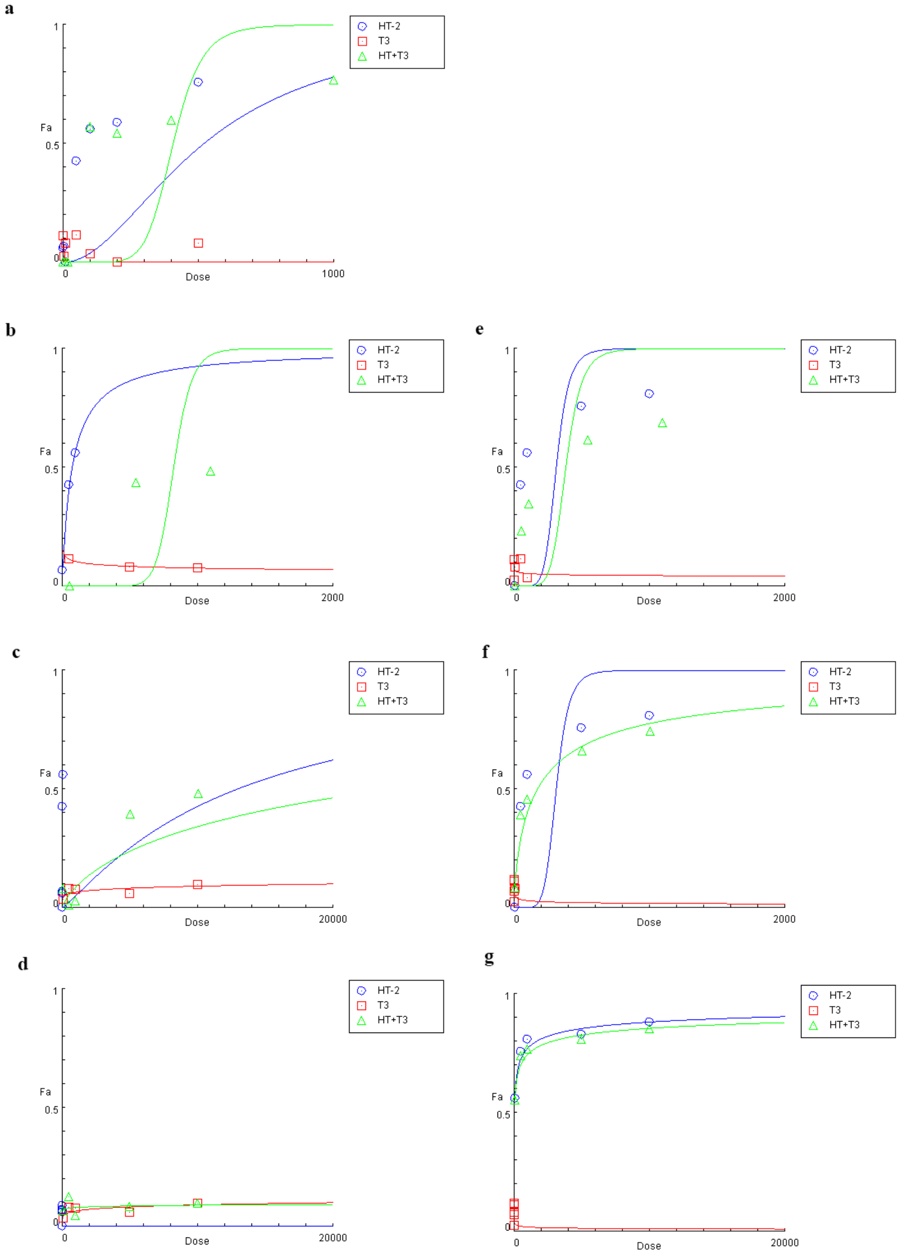

Figure S1. The dose-effect plot for figure 2 generated by CompuSyn software. Fa represents inhibition rate. The concentration ratios of HT-2:T3 were (**a**) 1:1, (**b**) 1:10, (**c**) 1:100, (**d**) 1:1000, (**e**) 10;1, (**f**) 100:1 and (**g**) 1000:1. The amounts of T3 and HT-2 toxin for each mixture are given in Table 1.
